# *PMS2* Pathogenic Variant in Lynch Syndrome-Associated Colorectal Cancer with Polyps

**DOI:** 10.1055/s-0042-1759888

**Published:** 2023-01-11

**Authors:** Henriette Poaty, Lauria Batamba Bouya, Aimé Lumaka, Arnaud Mongo-Onkouo, Deby Gassaye

**Affiliations:** 1 Embryology and Genetic Laboratory, Faculty of Health Sciences, Marien Ngouabi University, Brazzaville, Congo; 2 Department of Clinical Sciences, Institute of Research on Health Sciences, Brazzaville, Congo; 3 Centre de Génétique de l'Université de Kinshasa, DR Congo; 4 Service de Génétique Humaine, Sart Tilman, Avenue de l'Hôpital 13, 4000, Liège, Belgium; 5 Gastro-Enterology and Internal Medicine Service, CHU Brazzaville, Congo

**Keywords:** lynch syndrome, *PMS2* deficiency, mismatch repair gene, colorectal cancer

## Abstract

**Background** Lynch syndrome (LS) is an autosomal dominant condition due to the germline mutation in the mismatch repair (MMR) genes including *MLH1* , *MSH2* , *MSH6,* and *PMS2* (post-meiotic segregation increased 2). The MMR mutation carriers have high risk for cancers. Pathogenic *PMS2* variants are rarely reported in LS-associated colorectal cancer (CRC) with colorectal polyps. The aim of the study was to investigate the genetic etiology of CRC in an individual with CRC with multiple colorectal polyps and a family history of cancers.

**Patients and Methods** The index patient was an African male affected by CRC with multiple colorectal polyps. The clinical diagnostic for LS was based on the Amsterdam II criteria and pedigree. Next-generation sequencing with inherited cancer genes panel was used to detect the pathogenic variant.

**Results** The patient fulfilled the Amsterdam II criteria and the pedigree revealed a family history of recurrent CRC. A deleterious *PMS2* germline heterozygous mutation c.2192_2196delTAACT was detected.

**Conclusion** Our study supports the notion that LS may be associated with polyps and shows the predisposition of *PMS2* heterozygous mutation in LS-associated CRC at young age.

## Introduction

Lynch syndrome (LS) is an autosomal dominant disorder due to germline pathogenic variants in DNA mismatch repair (MMR) genes including *MLH1* , *MSH2* , *MSH6,* and post-meiotic segregation increased 2 ( *PMS2* ). 1 2 3 4 The *EpCam,* a neighboring gene of MSH2, is also responsible for LS. Classically, the condition is not associated with polyps.

The carriers of MMR deficiency are at high risk for gastrointestinal cancers, particularly colorectal cancer (CRC) and other primary malignancies in diverse sites at young age (endometrial, ovaries, brain, lung, skin, small bowel, pancreas, and cervix). 1 3 5 The Amsterdam criteria and Bethesda guidelines are useful clinical tools, for the identification of individuals and families at high risk of LS. 6 7

Deleterious variants in *PMS2* may be monoallelic (heterozygous) in LS or biallelic (homozygous) in constitutional MMR deficiency syndrome. 8 9 In comparison with others MMR genes *(MLH1* , *MSH2* , *MSH6* ), there is less data reported on *PMS2* mutations thus far. Here, we report one young African individual affected by CRC with colorectal polyps and a *PMS2* germline pathogenic variant.

## Patient and Methods

### Patient

The proband is a 51 year-old-Congolese male, recruited at the Gastroenterology and Cancerology Services of the Brazzaville Teaching Hospital, Congo in the frame of a research study hereditary on CRC in Brazzaville. 6 10

He had personal medical problems evolving for 8 years. It all started with diarrhea and abdominal pains, followed by rectal bleeding, significant weight loss, and anemia.

### Methods

The study was performed based on Amsterdam II clinical criteria for LS, pedigree to detect a family history of cancers, colonoscopy examination, and anatomical–pathological analysis in search of polyps and to confirm the malignancy. The final diagnosis was provided by DNA sequencing to identify the germline mutation.

Genomic DNA was extracted from peripheral lymphocytes, using the magnetic beads technique on Chemagic 360 from PerkinElmer. A gene panel of 39 genes involved in hereditary CRC resulting from polyposis or nonpolyposis hereditary syndromes was sequenced in PE100 on a HiSeq (Illumina, Inc, San Diego, California, United States) in Ogenetics facility. Library was prepared using the Oto Inherited Cancer panel kit (Ogenetics Inh Ca). The panel includes the following genes *: BMPR1A* , *SMAD4, STK11, PTEN, APC, MUTYH, NF1, MLH1, MSH2, MSH6, PMS2, EPCAM, RET, ATM, BARD1, BRCA1, BRCA2, TP53, BRIP1, CDH1, CDK4, CDKn2A, CHEK2, ELAC2, FANCC, HRAS1, MEN1, MET, NBN, NTRK1, PALLD, PTCH, RAD50, RAD51, RAD51C, RAD51D, VHL, MRE11A, PALB2* .

Hundred percent of the target was covered with >600 reads. A vcf file was returned and analyzed with Moon (Diploid, Heverlee, Belgium), using cancer (HP:0002664) and intestinal polyposis (HP:0200008) as HPO terms.

The study required the Ethics Committee approval (Medical Congo Ethics Commission approval, 00171/DGRST/CERSSA).

## Results

**Fig. 1:**
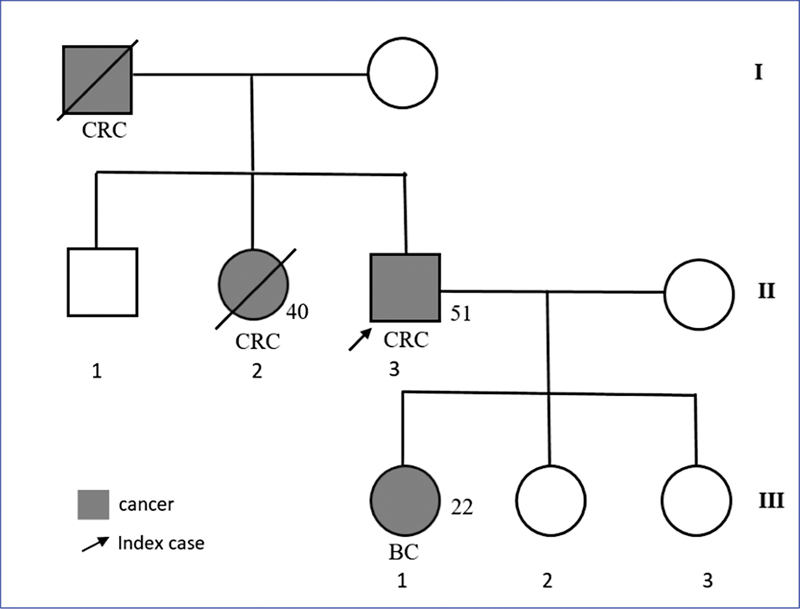
*Family pedigree of the index patient with a deleterious PMS2 mutation. (c.2192_2196delTAACT).* Note three successive affected generations (vertical transmission). The index patient had colorectal cancer (CRC) at the age of 51 years and three family members also had cancer. His daughter (first-degree) 22-years old had breast cancer (BC). His sister at the age of 40 years, and his father (first-degree) died from CCR. CCT, clinical complete response.

**Fig. 2:**
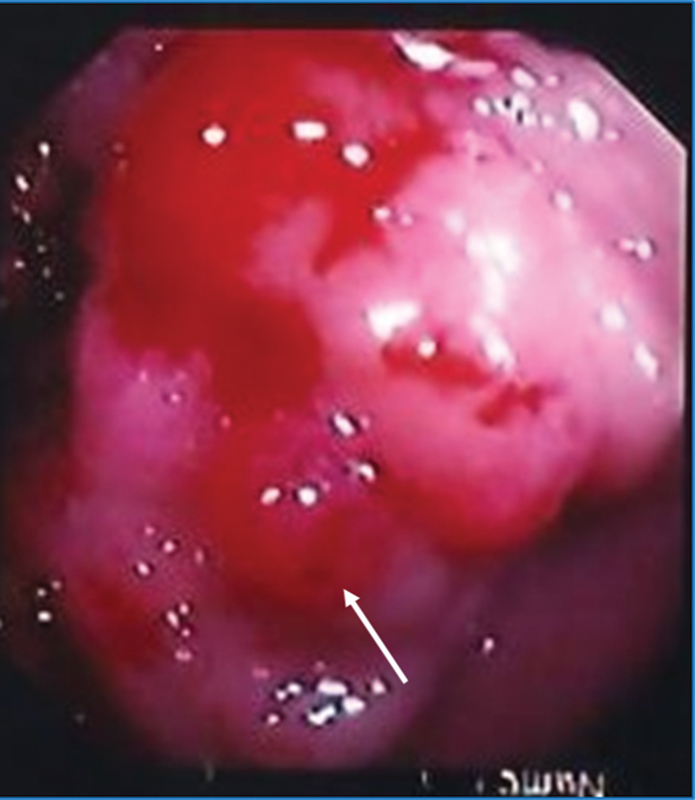
*Macroscopic tumor per colonoscopy* . Note hemorrhagic appearance of the tumor and presence of colonic polyps.

The phenotypic pedigree ( Fig. 1 ) revealed a family history of cancers. His daughter had breast cancer, whereas his sister and father died from CRC. Colonoscopy showed ulcero-budding, infiltrating, and hemorrhagic lesion ( Fig. 2 ), with multiple sessile polyps located on the left side of the colon and rectum. Histopathological analysis performed on a tumor biopsy diagnosed a colorectal adenocarcinoma. The patient fulfilled the Amsterdam II criteria.

**Fig. 3:**
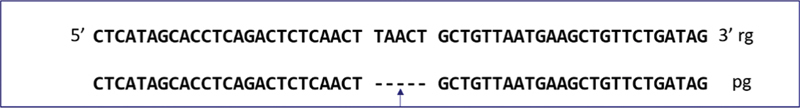
*PMS2 mutation.* Mutation in exon 13 of *PMS2* by deletion of five dinucleotides: thymine (T), adenines (A), cytosine (C), at position 2192 to 2196, defined as « c.2192_2196delTAACT ». This mutation is at the origin of a predictive pathogenic variant « p. Leu731Cysfs*3 »: codon substitution leucine position 731 in cysteine and premature stop codon, 3 amino acids later the substitution. rg, reference genome; pg, patient genome.

Next Generation sequencing of multigene panel identified a heterozygous pathogenic frameshift variant in *PMS2,* NM_000535.7: c.2192_2196delTAACT; *p* . Leu731Cysfs*3 (rs63750695) ( Fig. 3 ), a variant previously described in LS patients [PMID: 26845104, PMID: 20186688, PMID: 15872200, PMID: 15256438, PMID: 16472587, PMID: 24033266].

We concluded to LS-associated CRC with a pathogenic *PMS2* mutation. The patient underwent chemotherapy and surgical cure. Two years later, he left for Europe.

## Discussion

*PMS2* gene is located in 7p22.1 chromosomal segment with 38,125 base pairs, 15 exons and the encoded protein contains 862 amino acids (GRCh38/hg38). 11 *PMS2* protein contains ATPase and endonuclease domains. The last domain acts with *MLH1* to give a protein complex that interacts with other proteins to repair DNA errors during replication.

Heterozygous mutation in the *PMS2* gene (MIM 600259) are responsible for hereditary nonpolyposis CRC-4 (HNPCC4) (MIM 614337), which is a form of LS. This syndrome is the most common hereditary form of clinical complete response (CCR) due to inherited mutations in the MMR genes.

### LS and Polyps

Individuals with LS can have polyps in their life, 12 13 and the sessile-type adenomatous polyps as observed in our case predispose to the dysplasia and cancer. 14

The polyps, located on all sites of colon increase the risk of CRC and they are associated with MMR gene deficiencies including *PMS2* mutations (OMIM#614337). 8 12 The localization of the polyps in our patient was consistent with those previous reports. In contrast, our patient had more than 10 polyps at the time of diagnostic, which is higher than the number usually reported, less than 10.

### *PMS2* Mutations

Globally, *PMS2* mutations cause LS in approximately 8 to 15% of cases. 5 11 15 That incidence is variable according to the countries and methods performed to establish diagnosis of LS: PCR, microsatellite instability and IHC or DNA sequencing. It should be noted than some of the *PMS2* mutations are recurrent. 3 15 16 17

The heterozygous *PMS2* mutations are known to be responsible for LS in 5 to 15% of cases. 18 19 However, in one study looking at 61 LS due to *PMS2* deficiency, heterozygous *PMS2* mutations occurred in 90.16% (55/61) while the homozygous *PMS2* mutations occurred in 9.83% (6/61) of LS. 8

The heterozygous mutation c.2192_2196delTAACT detected in our patient has been already identified in African Americans and in Caucasian Americans patients at young age. 8 20 21 22 It is predicted to cause the substitution of the amino acid leucine 731 and the premature termination of the protein 3 amino acids downstream, p. Leu731Cysfs*3. This variant is predicted to cause loss of normal function through truncated protein. This mutation is one of the *PMS2* pathogenic variants listed in the International Society for Gastrointestinal Hereditary Tumors (InSiGHT) database ( http://www.insight database.org/classifications /gene). The colonic cancer caused by this pathogenic variant is often located in transverse or left colon. 8 20 21

### *PMS2* Cancer Predispositions

In LS, the risk for any cancer by the age of 70 years is 63.7% and the syndrome is responsible of 2 to 5% of all CRCs. 2 15

Regarding cancer predisposition in *PMS2* mutation carriers, cumulative risk for any cancer in both sex is approximately 10% at the age 50 to 55 years. 23 Various types of cancers are observed: CRC, endometrial, ovarian, prostate, lung, brain, kidney pancreas, skin, small bowel, cervix. 1 4 11 18 24 25 CRC risk is 5.2 times higher than in healthy persons. The age-related risk for CCR is 22% for persons in their 50s. 11 One cohort study reported that the most frequent cancer in *PMS2* mutations was CRC (in 80% of cases) followed by endometrial cancer (in 8.1% of cases). 18

As observed in our pedigree, *PMS2* deficiency is also associated with increased risk of breast cancer and cumulative risk at the age of 60 years is 37.7%. 4 Sheehan et al 26 reported an increased prevalence of breast cancer among women carrying LS due to mutations in *PSM2* as compared with those carrying mutations in *MLH1* , *MSH2* , and *MSH6* genes.

In our patient, family history is compatible with cancer predisposition with the sister and father affected with CRC and his daughter with breast cancer.

The Table 1 reports some recurrent *PMS2* deleterious mutations detected in individuals (of diverse populations) affected by LS-associated cancers, mainly the CCR.

**Table 1:** Examples of recurrent PMS2 pathogenic variants in individuals with cancers (ClinVar)

Nucleotide change	Protein change	E	Cancers site	Age Ds /Gender	Countries	References
c.736_741del6ins11	p.Pro246_Pro247 delinsCysValTer	7	Colon, Cecum, Rectosigmoid, Stomach	28–74; M, F	United States Australia	8; 15; 19
c.903G > T	p.Lys301Asn	8	Colon, Endometrium	54–61; M, F	United States	8
c.862_863del	p.Gln288fs	8	Colon	54	United States	19
c.949C > T	p.Gln317Ter	9	Colon, Brain	39		3; 8
c.943C > T	p.Arg315Ter	9	Ovaries	F	Chine	24
c.904_1144del		9	Colon, Cecum, Rectum	M, F	Australia	15
c.137G > T	p.Ser46Ile	10	Small bowel, Transverse colon, Cecum, Sigmoid	32–67; M, F	France United States	3; 8; 17
Exon 10 deletion		10	Colon, Cecum, Rectum, Endometrium	30–60; M, F	United States	8; 19
c.1021delA	p.Arg341fs	10	Esophagium, Colon, Rectum, Breast, Skin, Endometrium	45–66; M, F	Australia	16
c.2444C > T	p.Ser815Leu	14	–	–	Netherlands	18; 19
c.903 + 1G > A		8	Colon, Cecum, Rectum	32–40; M, F	United States	19
c.1500del	p.Val501TrpfsTer94	11	Colon, Brain Ovaries, Endometrium	10–37; F	Pakistan	9
c.2182_2184delACTinsG	p.Thr728Alafs	13	Colon, Rectum	F, M	United States	8; 14
c.2192_2196delTAACT	p.Leu731Cysfs*3	13	Transverse colon	22–23; M	United States	8; 19; 20
			Left colon, Rectum	51; M	Congo	Present study

Abbreviations: Age Ds, age of diagnosis (years); E, exon; F, female; M, male.

## Conclusion

Our report and published papers underline the notion that LS is also associated with multiple polyps. In addition, our data in accordance with the review highlights that the recurrent heterozygous *PMS2* pathogenic variants can be identified in diverse patient populations (Caucasian, Africa). They are associated with an increased risk for CRC at young age. This report also supports the need for more study into the association between LS and breast cancer, especially in African patients.

## What Does This Article Bring New?

First, we report the first Central African patient with a known LS-associated pathogenic variant, who also has multiple colonic polyps.
Second, our results provide additional information about the clinical phenotype of the deleterious *PMS2* mutation « c.2192_2196delTAACT » that contributes to colorectal carcinogenesis in Caucasian and also in African populations.
